# Donor-Recipient Age Mismatch and Long-Term Graft Outcomes After Adolescent Liver Transplant

**DOI:** 10.1001/jamanetworkopen.2025.52779

**Published:** 2026-01-07

**Authors:** Toshihiro Nakayama, Amanda R. Jensen, Antony Attia, Daniel J. Ahn, Daniel J. Firl, Allison Kwong, Vivek Charu, Marc L. Melcher, Carlos O. Esquivel, Kazunari Sasaki

**Affiliations:** 1 Stanford Transplant Outcomes Research Center (STORC), Division of Abdominal Transplantation, Stanford University Medical Center, Stanford, California; 2 Division of Abdominal Transplantation, Department of Surgery, Stanford University Medical Center, Stanford, California; 3 Department of Surgery, Duke University Medical Center, Durham, North Carolina; 4 Division of Gastroenterology and Hepatology, Department of Medicine, Stanford University, Redwood City, California; 5 Department of Pathology and Quantitative Science Unit, Stanford University, Stanford, California

## Abstract

**Importance:**

Donor-recipient age mismatch is an established risk factor in adult liver transplants (LTs), yet its effect in adolescents, who require long-term graft durability, has not been fully characterized. Despite pediatric prioritization, some adolescent donor livers are allocated to adults, limiting access to age-matched grafts for adolescents.

**Objective:**

To assess whether a donor-recipient age difference of 10 or more years is associated with inferior graft survival in LTs among adolescents and to estimate the benefits of broader geographic sharing of adolescent donor livers.

**Design, Setting, and Participants:**

This retrospective, registry-based case-control study used data from the Organ Procurement & Transplantation Network database, a nationwide US transplant registry. Participants were adolescents aged 12 to 17 years who received liver-only grafts from donation after brain death between March 1, 2002, and December 31, 2024, with follow-up until April 4, 2025. Propensity score matching (1:1) was performed on graft type and size mismatch, donor sex, donor-recipient sex mismatch, transplant center volume, and recipient variables.

**Exposure:**

Donor-recipient age difference of 10 or more years (age-mismatched graft) vs less than 10 years (age-matched graft).

**Main Outcomes and Measures:**

The primary outcome was 10-year graft survival. The secondary outcome was 10-year overall survival. Waiting time to an age-matched graft offer under alternative donor-sharing radii (1500 nautical miles [NM], 1000 NM, or no limit vs 500 NM) were also estimated.

**Results:**

Among 2020 adolescents receiving LTs (median age, 15.0 [IQR, 13.0-16.0] years; 1081 [53.5%] female), 612 (30.3%) received age-mismatched grafts (median donor age, 36.0 [IQR, 29.0-45.0] years) and 1408 (69.7%) received age-matched grafts (median donor age, 16.0 [IQR, 13.0-17.0] years). The age-mismatched group had a higher proportion of recipient candidates in the intensive care unit at transplant (287 [46.9%] vs 250 [17.8%]; *P* < .001). After propensity score matching (n = 526 per group), 10-year graft survival was 61.5% in the age-mismatched group and 74.2% in the age-matched group (*P* < .001), with consistent results across recipients’ pretransplant hospitalization status. A simulation estimated that expanding the adolescent allocation radius to 1000 NM would allow 90% of adolescent candidates to receive age-matched offers within 15 days, compared with 44 days under the current 500-NM limit.

**Conclusions and Relevance:**

In this case-control study of a US national cohort of adolescents receiving LT, donor-recipient age mismatch of 10 or more years was associated with inferior graft survival. Broader allocation of adolescent donors may improve access to age-matched grafts and long-term outcomes.

## Key Points

**Question:**

Among adolescent liver transplant recipients, is receiving from a donor 10 or more years older associated with worse long-term graft survival?

**Findings:**

In a nationwide case-control study of a registry cohort of 2020 adolescent recipients of liver transplants, 30% received age-mismatched grafts (donor ≥10 years older than recipient). After propensity score matching, age-mismatched transplants had inferior 10-year graft survival than when donors were less than 10 years older than recipients (62% vs 74%).

**Meaning:**

These findings suggest the need for policy adjustments such as broader sharing of adolescent donor livers to improve long-term graft survival for adolescent recipients.

## Teaser

This case-control study compares long-term graft survival outcomes of adolescents receiving liver transplants from donors 10 or more years vs less than 10 years older.

## Introduction

Liver transplant (LT) is the optimal treatment for end-stage liver disease. In an era of persistent organ shortage, finding a suitable donor for the recipient is challenging but also paramount to the success of LT.^1,2^ One important matching criterion is age. Donor age is a well-established prognostic factor for both short- and long-term graft survival.^3,4^ In adult LT, the adverse impact of older donor age on graft outcomes is most pronounced in recipients under 40 years of age. In contrast, in recipients older than 60 years, donor age appears to have little effect. These age-related differences persist for more than a decade posttransplant.^5,6^

In the US, under the Acuity Circles policy, pediatric livers are preferentially allocated to pediatric recipients within 500 nautical miles (NM) of the donor hospital, not only to reduce waiting list mortality but also in recognition of ethical frameworks such as the prudential lifespan account (which argues for investing resources where they yield the greatest lifetime benefit) and the fair innings argument (which holds that everyone deserves a chance at a full lifespan).^7,8,9,10^ Despite this policy, many adolescent liver grafts are allocated to adults when no eligible pediatric candidates within 500 NM are available to accept the offer. A pediatric waiting list mortality rate of 0 has yet to be achieved, and the supply of pediatric donors remains substantially smaller than that of adults.^11^ Consequently, grafts from young adult donors (aged in their 20s or 30s) are often used for adolescent recipients, whose body size is comparable to that of young adults. However, the long-term effects of this donor-recipient age mismatch in adolescent LT candidates, whose life expectancy is longer than that of young adults, have not been clearly defined.

Because the wait for an age-matched graft offer is unpredictable, accepting an age-mismatched graft can be a rational choice, especially for sicker recipients. We speculated that broader offering of adolescent livers beyond 500 NM might alleviate the need for age-mismatched transplants. The aims of this study were to evaluate the association of donor-recipient age mismatch with long-term graft survival for adolescents and estimate whether broader sharing may influence waiting time for age-matched offers.

## Methods

### Design and Cohort Selection

This case-control study used the Organ Procurement & Transplantation Network (OPTN) Standard Transplant Analysis and Research (STAR) file supplemented by donor and recipient center data. The cohort consisted of adolescents aged 12 to 17 years who underwent liver-only transplant from donation after brain death (DBD) between March 1, 2002, and December 31, 2024, excluding multiorgan and living donor transplants. Candidates younger than 12 years were excluded because the allocation system differs and the frequent use of split or partial grafts might introduce confounding.^12^ LTs from donation after circulatory death (DCD) were excluded due to small volume. The last day of follow-up was April 4, 2025. All the analyses were conducted with the approval of the institutional review board at Stanford University, which granted a waiver of informed consent because data were deidentified. This study was performed according to the Strengthening the Reporting of Observational Studies in Epidemiology (STROBE) reporting guideline.^13^

### Setting and Exposure

The OPTN STAR file is a nationally representative database maintained by the United Network for Organ Sharing, which includes all US transplant centers. The exposure was donor-recipient age mismatch, defined as a difference of 10 or more years between donor age and recipient age. We chose this cutoff because it included a larger proportion of the cohort than an age difference of 20 years or more, thus providing more common clinical scenarios.

### Outcomes

The primary outcome was 10-year graft survival. The secondary outcome was 10-year overall survival. Subgroup analyses stratified recipients by pretransplant hospitalization status and transplant year (before vs after 2010).

### Covariates

The following variables were extracted from the database: split or reduced graft, donor characteristics (age, sex, height, and weight), recipient characteristics (age, sex, height, weight, diagnosis, Model for End-Stage Liver Disease [MELD] score [range, 6-40, with higher scores indicating higher risk of death within 90 days], status 1 [most urgent medical priority], previous transplant, and pretransplant admission status [intensive care unit (ICU), hospital wards, or home]), cold ischemia time, and donor and recipient hospital addresses. Donor-recipient graft size mismatch was defined by the body surface area (BSA) index, or the ratio of donor to recipient BSA.^2,14,15^ BSA was calculated using height and weight at transplant by the Du Bois formula.^16^ Size mismatch was defined as small (BSA index <0.78), normal (0.78-1.24), or large (>1.24), as described previously, and was not defined for split or reduced grafts.^2,14^ Center volume was grouped into tertiles of overall cases per center during the study period: high (≥57 cases), middle (28-56 cases), and low (<28 cases). Donor and recipient hospital addresses were used to calculate interhospital distance for the counterfactual reallocation.

### Statistical Analysis

Donor and recipient demographics were presented as frequencies with percentages or median values with IQRs. Differences between categorical values were assessed using the χ^2^ test, while continuous values were analyzed using the Mann-Whitney *U* or Kruskal-Wallis test, as appropriate. We also summarized temporal trends and between-center variation in the use of age-mismatched donors.

We performed 1:1 nearest-neighbor propensity score matching on graft type and size mismatch (split or reduced graft or small-sized, normal-sized, or large-sized whole-liver graft), donor sex, donor-recipient sex mismatch, transplant center volume, and the following recipient variables: age, sex, BSA, diagnosis, laboratory MELD score at transplant, status 1 at transplant, and pretransplant admission status (ICU, hospital wards, or no admission). The caliper was set at 0.1. Graft survival was evaluated using the Kaplan-Meier method and log-rank tests and was measured from transplant until retransplant or death. Patients without events were censored at 10 years, last recorded follow-up, or administrative censoring on April 4, 2025, whichever came first. Multivariable Cox proportional hazards regression models estimated the hazard ratios (HRs) with 95% CIs of LTs for grafts from age-mismatched donors, adjusting for graft type and size mismatch, donor and recipient sex and sex mismatch, recipient BSA, recipient diagnosis, laboratory MELD score, status 1, cold ischemia time, and pretransplant hospitalization status. For Kaplan-Meier analysis and Cox proportional hazards regression, we performed landmark analyses at 1 year and 5 years after transplant.^17,18^ We redefined time 0 at each landmark, including only recipients alive with a functioning graft and receiving follow-up, to separate early and late risk and to limit confounding from unmeasured illness severity that may influence offer acceptance.

Additionally, to explore if broader allocation of adolescent donors might help reduce age-mismatched transplants, we used a retrospective counterfactual reallocation of age-mismatched recipients to the next age-matched donor in the observed timeline. For every age-mismatched LT (index date = day 0) performed on or after February 4, 2020 (Acuity Circles policy implementation), we identified adolescent DBD grafts transplanted into adults on days 1 to 90 whose donor weight was within 20 kg of the index recipient’s weight. Split grafts were excluded. Marked size mismatch was excluded because adolescent recipients vary widely in body size.^19,20^ We reassigned these adolescent grafts to the adolescent recipients who had accepted age-mismatched grafts. Four travel-distance ceilings were modeled—500 NM, 1000 NM, 1500 NM, and no limit. For each scenario, we calculated the 90th percentile wait time, the day by which 90% of individuals would have received an adolescent graft offer. For example, a time of 14 days means that no adult transplant using an adolescent graft occurred on days 1 to 14 in the remaining 10% of individuals. As a supplementary analysis, donor and recipient characteristics were compared between adult recipients of adolescent grafts and adolescents receiving age-matched livers. Statistical significance was established below a 2-sided *P* value of .05. Statistical analyses were conducted using R, version 4.4.0 (R Project for Statistical Computing).^21^

## Results

### Baseline Donor and Recipient Characteristics

**Figure 1.**
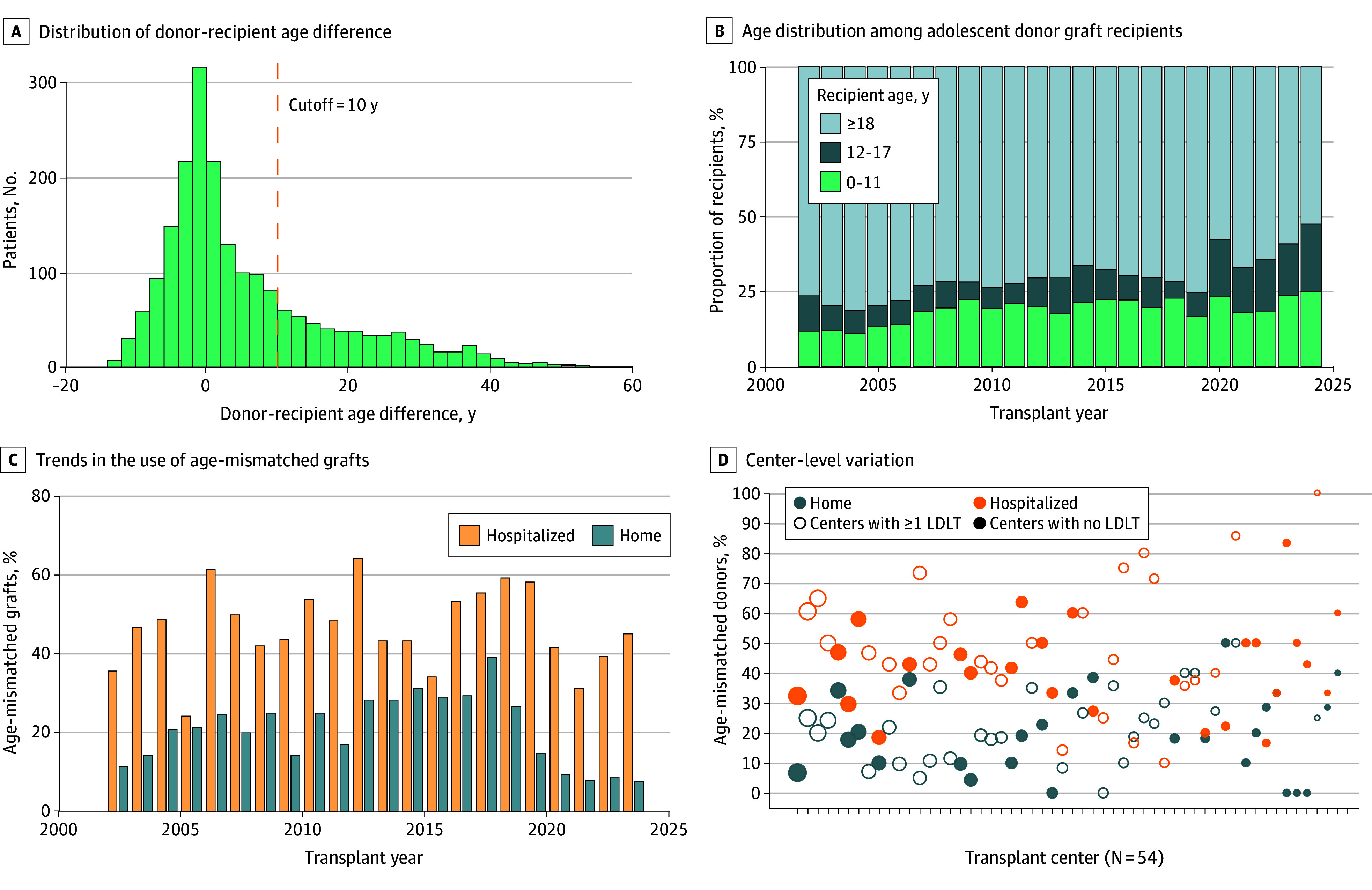
Trends in the Use of Age-Mismatched Grafts in Liver Transplant (LT) D, Centers are ordered by their adolescent LT volume, and marker size reflects that volume. LDLT indicates living donor LT.

Among 2020 adolescents receiving LTs (median age, 15.0 [IQR, 13.0-16.0] years; 1081 [53.5%] female, 939 [46.5%] male), 612 transplants (30.3%) involved a donor-recipient age mismatch (age difference, ≥10 years) and 1408 (69.7%) were age-matched (age difference, <10 years). Figure 1A shows the distribution of donor-recipient age difference. In the cohort before propensity score matching, donors in the age-mismatched group were older (median age, 36.0 [IQR, 29.0-45.0] years vs 16.0 [IQR, 13.0-17.0] years in the age-matched group; *P* < .001), and recipient age was similar (Table 1). In the age-mismatched group, 46 recipients (7.5%) received grafts from donors at least 40 years older. Recipients in the age-mismatched vs age-matched group had a higher proportion of sicker candidates, such as patients with acute liver failure (163 [26.6%] vs 180 [12.8%]; *P* < .001), status 1 at LT (329 [53.8%] vs 320 [22.7%]; *P* < .001), and admission into the ICU before LT (287 [46.9%] vs 250 [17.8%]; *P* < .001). Median waiting time was shorter in the age-mismatched group (18.0 [IQR, 3.0-112.8] days vs 68.0 [IQR, 14.0-221.2] days; *P* < .001). After propensity score matching (n = 526 in each group), donor and recipient characteristics were well balanced, with an absolute standardized mean difference of less than 0.1 (Table 1).

**Table 1.** Baseline Donor and Recipient Characteristics for Adolescent LTs^a^

Characteristic	Before matching				After matching			
	Age difference, y		*P* value	SMD	Age difference, y		*P* value	SMD
	<10 (n = 1408)	≥10 (n = 612)			<10 (n = 526)	≥10 (n = 526)		
Graft type and size								
Reduced or split graft	95 (6.7)	55 (9.0)	<.001	0.337	47 (8.9)	48 (9.1)	.83	0.058
Whole liver, small size	143 (10.2)	15 (2.5)			12 (2.3)	15 (2.9)		
Whole liver, normal size	965 (68.5)	429 (70.1)			362 (68.8)	368 (70.0)		
Whole liver, large size	205 (14.6)	113 (18.5)			105 (20.0)	95 (18.1)		
Donors								
Age, y	16.0 (13.0-17.0)	36.0 (29.0-45.0)	<.001	2.744	17.0 (15.0-20.0)	35.0 (28.0-43.8)	<.001	2.535
Sex								
Female	510 (36.2)	317 (51.8)	<.001	0.318	264 (50.2)	252 (47.9)	.50	0.046
Male	898 (63.8)	295 (48.2)			262 (49.8)	274 (52.1)		
Height, cm	166.7 (154.9-175.0)	168.0 (162.6-175.3)	<.001	0.435	167.6 (160.0-175.3)	168.0 (162.6-177.6)	.03	0.203
Weight, kg	61.2 (49.9-72.0)	68.0 (60.0-77.8)	<.001	0.581	64.3 (55.0-74.4)	68.0 (60.0-77.8)	<.001	0.328
BSA, m^2^	1.7 (1.5-1.9)	1.8 (1.6-1.9)	<.001	0.570	1.7 (1.6-1.9)	1.8 (1.6-1.9)	<.001	0.313
Donor-recipient sex mismatch	680 (48.3)	300 (49.0)	.80	0.014	265 (50.4)	251 (47.7)	.42	0.053
CIT, h	6.8 (5.2-8.6)	6.3 (5.0-8.0)	.01	0.079	7.0 (5.3-8.5)	6.3 (5.0-8.0)	.01	0.099
Recipients								
Age, y	15.0 (13.0-16.0)	15.0 (13.0-16.0)	.63	0.021	15.0 (14.0-16.0)	15.0 (13.0-16.0)	.04	0.123
Sex								
Female	754 (53.6)	327 (53.4)	>.99	0.002	293 (55.7)	281 (53.4)	.50	0.046
Male	654 (46.4)	285 (46.6)			233 (44.3)	245 (46.6)		
Height, cm	160.0 (152.4-168.0)	162.6 (154.9-170.1)	<.001	0.181	162.0 (153.5-169.0)	162.6 (154.9-170.1)	.33	0.045
Weight, kg	55.3 (45.0-66.2)	57.8 (47.6-70.5)	<.001	0.202	56.7 (46.8-68.8)	57.4 (46.5-70.0)	.74	0.039
BSA, m^2^	1.6 (1.4-1.7)	1.6 (1.4-1.8)	<.001	0.209	1.6 (1.4-1.8)	1.6 (1.4-1.8)	.55	0.041
Diagnosis								
Biliary atresia	134 (9.5)	25 (4.1)	<.001	0.446	20 (3.8)	25 (4.8)	.91	0.063
Acute liver failure	180 (12.8)	163 (26.6)			122 (23.2)	128 (24.3)		
Metabolic	168 (11.9)	88 (14.4)			68 (12.9)	66 (12.5)		
Other								
Cholestatic^b^	253 (18.0)	63 (10.3)			66 (12.5)	60 (11.4)		
Noncholestatic^c^	673 (47.8)	273 (44.6)			250 (47.5)	247 (47.0)		
MELD score^d^	16.0 (11.0-22.0)	25.0 (14.0-32.0)	<.001	0.661	21.0 (14.0-29.0)	22.0 (13.0-30.0)	.67	0.033
Status 1^e^	320 (22.7)	329 (53.8)	<.001	0.674	235 (44.7)	250 (47.5)	.39	0.057
Pretransplant condition								
ICU	250 (17.8)	287 (46.9)	<.001	0.697	203 (38.6)	218 (41.4)	.57	0.065
Hospital	187 (13.3)	88 (14.4)			83 (15.8)	74 (14.1)		
Home	971 (69.0)	237 (38.7)			240 (45.6)	234 (44.5)		
History of previous transplant	149 (10.6)	71 (11.6)	.55	0.032	65 (12.4)	60 (11.4)	.70	0.029
Transplant center volume during study period^f^								
High (≥57 cases)	499 (35.4)	202 (33.0)	.36	0.069	180 (34.2)	171 (32.5)	.84	0.036
Middle (28-56 cases)	469 (33.3)	200 (32.7)			168 (31.9)	173 (32.9)		
Low (<28 cases)	440 (31.2)	210 (34.3)			178 (33.8)	182 (34.6)		
Waiting time, d	68.0 (14.0-221.2)	18.0 (3.0-112.8)	<.001	0.221	27.0 (5.0-133.8)	29.0 (4.0-148.0)	.90	0.003

Abbreviations: BSA, body surface area; CIT, cold ischemia time; ICU, intensive care unit; LT, liver transplant; MELD, Model for End-Stage Liver Disease; SMD, standardized mean difference.

^a^ Data for continuous variables are presented as median (IQR) and for categorical variables as number (percentage).

^b^ Other cholestatic diagnoses include primary sclerosing cholangitis, primary biliary cirrhosis, biliary hypoplasia (including Alagille syndrome and nonsyndromic paucity of intrahepatic bile duct), familial cholestasis (including Byler disease), secondary biliary cirrhosis (including Caroli disease and choledochal cyst), and neonatal cholestatic liver disease.

^c^ Other noncholestatic diagnoses include cirrhosis (autoimmune, cryptogenic, chronic active hepatitis, viral hepatitis, drug or industrial exposure), malignancy (hepatocellular carcinoma, hepatoblastoma, fibrolamellar hepatocellular carcinoma, cholangiocarcinoma, and other primary or secondary malignant tumors), cystic fibrosis, vascular diseases (Budd-Chiari syndrome), congenital hepatic fibrosis, trauma, hyperalimentation-induced liver disease, and graft failure.

^d^ Score range, 6 to 40, with higher scores indicating higher risk of death within 90 days.

^e^ Indicates most urgent medical priority.

^f^ Of 103 total centers, 9 (8.7%) were high volume; 16 (15.5%), middle volume; and 78 (75.7%), low volume.

### Trends in Use of Age-Mismatched Deceased Donors for Adolescent Candidates

In 2024, over half of adolescent donor livers (152 of 290 [52.4%]) were used for adults (Figure 1B). The use of age-mismatched donors varied by recipient’s hospitalization status and era (Figure 1C). The proportion of age-mismatched LTs fluctuated between 8 of 33 (24.2%) and 18 of 28 (64.3%) for candidates hospitalized before transplant and dropped from 20 of 51 (39.2%) in 2018 to less than 10% after the 2020 policy change for candidates not hospitalized (eg, 5 of 65 [7.7%] in 2024). Center-level use of age-mismatched donors varied but was similar between centers with at least 1 living donor LT (LDLT) (28 [51.9%] of the 54 centers with ≥10 deceased donor LT cases in the study period) and those with no LDLTs (26 [48.1%] of the 54 centers with ≥10 deceased donor LT cases) (Figure 1D).

### Posttransplant Survival

**Figure 2.**
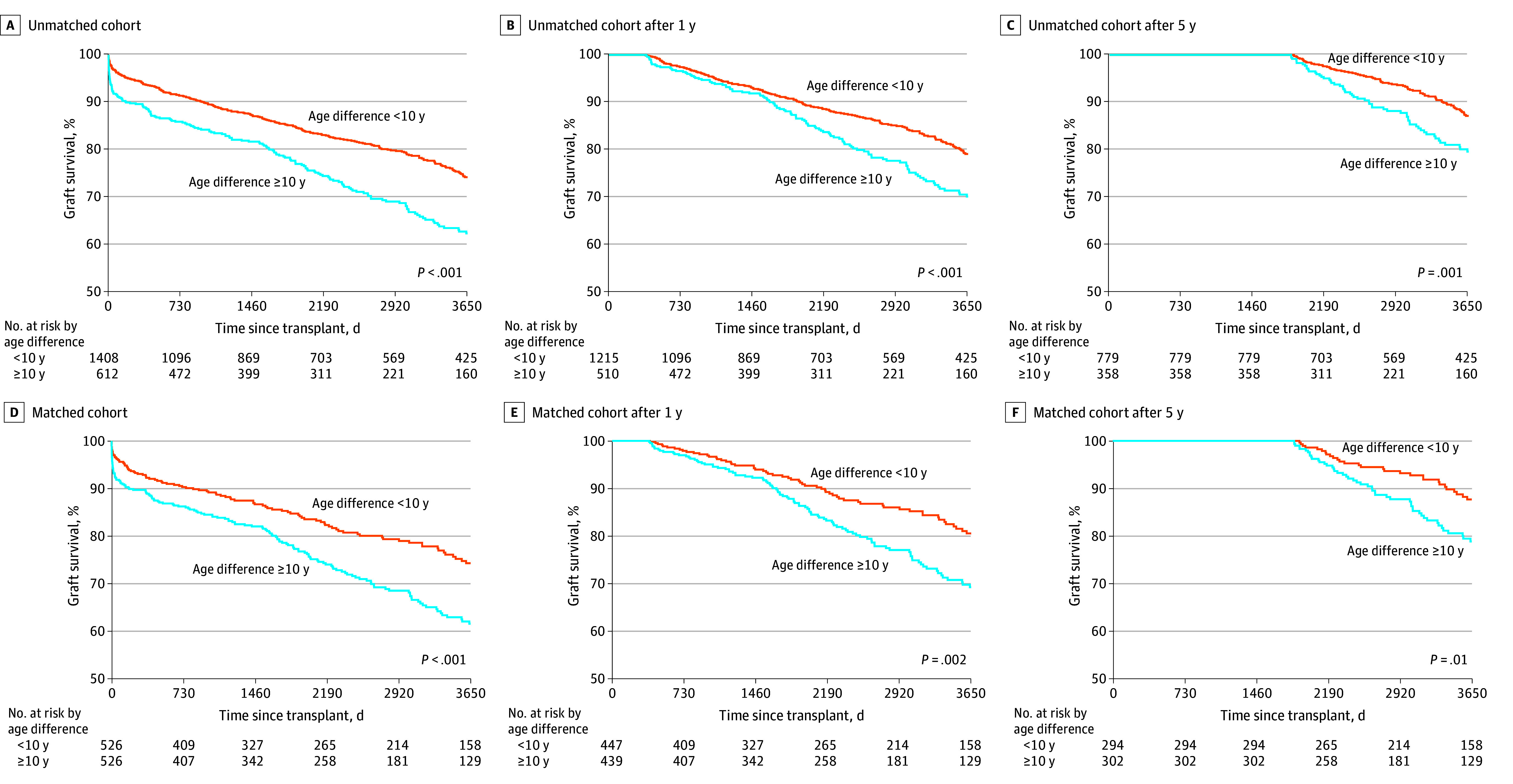
Kaplan-Meier Survival Curves in the Unmatched and Matched Cohorts

Median follow-up was 6.1 years (IQR, 2.2-11.0 years) in the cohort before propensity score matching and 6.1 years (IQR, 2.3-10.4 years) in the matched cohort. Age-mismatched LTs had significantly lower 10-year graft survival than age-matched LTs before (62.4% vs 74.2%; *P* < .001) and after (61.5% vs 74.2%; *P* < .001) propensity score matching (Figure 2A and D); the HR in the age-mismatched cohort was 1.39 (95% CI, 1.11-1.73; *P* = .004) (Table 2). In landmark analyses, after surviving 1 year, graft survival between cohorts was similar until 5 years (Figure 2B and E) but was worse beyond 5 years for the age-mismatched cohort (Figure 2C and F); the HR at 5 years in the age-mismatched cohort was 1.67 (95% CI, 1.12-2.48; *P* = .01) (Table 2).

**Table 2.** Cox Proportional Hazards Regression Analysis for 10-Year Graft Survival in the Cohort Before Propensity Score Matching

Variable	Overall cohort		Landmark at 1 y^a^		Landmark at 5 y^a^	
	HR (95% CI)	*P* value	HR (95% CI	*P* value	HR (95% CI	*P* value
Age difference ≥10y	1.39 (1.11-1.73)	.004	1.45 (1.11-1.90)	.01	1.67 (1.12-2.48)	.01
Graft type and size mismatch (vs whole liver, normal size)						
Split or reduced graft	1.62 (1.14-2.30)	.01	1.14 (0.71-1.84)	.31	1.28 (0.64-2.57)	.45
Whole liver, small size	1.49 (1.06-2.09)		1.44 (0.95-2.18)		1.44 (0.75-2.75)	
Whole liver, large size	1.27 (0.94-1.72)		1.23 (0.84-1.79)		1.44 (0.84-2.48)	
Male donor	0.81 (0.66-0.99)	.04	0.81 (0.63-1.04)	.09	0.96 (0.66-1.39)	.81
Donor-recipient sex mismatch	1.08 (0.88-1.31)	.47	1.02 (0.80-1.31)	.85	0.99 (0.69-1.43)	.96
Male recipient	1.10 (0.90-1.34)	.37	0.97 (0.76-1.23)	.78	0.98 (0.68-1.41)	.90
Recipient BSA, per 0.1 m^2^ increase	1.05 (1.00-1.09)	.04	1.07 (1.01-1.13)	.01	1.11 (1.02-1.20)	.01
Diagnosis (vs biliary atresia)						
Acute liver failure	1.72 (0.99-3.01)	<.001	2.22 (1.10-4.45)	<.001	1.56 (0.62-3.93)	.02
Metabolic	0.84 (0.45-1.58)		0.96 (0.44-2.07)		0.66 (0.22-1.92)	
Other cholestatic^b^	1.90 (1.10-3.25)		2.49 (1.29-4.81)		2.24 (0.96-5.23)	
Other noncholestatic^c^	2.11 (1.28-3.48)		2.37 (1.28-4.41)		1.73 (0.79-3.83)	
CIT ≥8 h	1.22 (0.99-1.52)	.06	1.07 (0.82-1.41)	.61	1.00 (0.67-1.50)	.98
MELD score, per point increase	1.01 (1.00-1.03)	.07	1.01 (0.99-1.03)	.38	1.03 (1.01-1.06)	.02
Recipient’s pretransplant condition (vs home)						
ICU	1.38 (0.95-2.02)	.24	1.18 (0.73-1.89)	.79	1.31 (0.67-2.55)	.41
Hospital	1.17 (0.86-1.60)		1.09 (0.75-1.58)		0.82 (0.45-1.49)	
Status 1^d^	0.82 (0.59-1.14)	.24	0.64 (0.42-0.98)	.04	0.38 (0.20-0.73)	.004
Center volume (vs high)						
Low	1.19 (0.94-1.51)	.02	1.27 (0.95-1.69)	.03	1.48 (0.96-2.26)	.06
Middle	0.84 (0.65-1.08)		0.84 (0.62-1.16)		0.90 (0.55-1.44)	

Abbreviations: BSA, body surface area; CIT, cold ischemia time; HR, hazard ratio; ICU, intensive care unit; MELD, Model for End-Stage Liver Disease.

^a^ Landmark risk sets included only recipients alive with a functioning graft and under follow-up at the landmark; time 0 indicates reset to the landmark, and events before the landmark were excluded.

^b^ Other cholestatic diagnoses include primary sclerosing cholangitis, primary biliary cirrhosis, biliary hypoplasia (including Alagille syndrome and nonsyndromic paucity of intrahepatic bile duct), familial cholestasis (including Byler disease), secondary biliary cirrhosis (including Caroli disease and choledochal cyst), and neonatal cholestatic liver disease.

^c^ Other noncholestatic diagnoses include cirrhosis (autoimmune, cryptogenic, chronic active hepatitis, viral hepatitis, drug or industrial exposure), malignancy (hepatocellular carcinoma, hepatoblastoma, fibrolamellar hepatocellular carcinoma, cholangiocarcinoma, and other primary or secondary malignant tumors), cystic fibrosis, vascular diseases (Budd-Chiari syndrome), congenital hepatic fibrosis, trauma, hyperalimentation-induced liver disease, and graft failure.

^d^ Indicates most urgent medical priority.

Graft survival findings for age-mismatched vs age-matched LTs were consistent by recipient’s hospitalization status at transplant before (hospitalized: 60.2% vs 69.3% [*P* = .02]; nonhospitalized: 65.6% vs 76.5% [*P* = .002]) and after (hospitalized: 57.9% vs 70.0% [*P* = .02]; nonhospitalized: 65.6% vs 79.2% [*P* = .003]) propensity score matching (eFigure 1 in Supplement 1) and by era before (2002-2010: 54.5% vs 69.4% [*P* < .001]; 2011-2024: 69.1% vs 79.7% [*P* = .002]) and after (2002-2010: 52.5% vs 67.3% [*P* = .02]; 2011-2024: 68.4% vs 82.1% [*P* = .01]) propensity score matching (eFigure 2 in Supplement 1). Ten-year overall survival was lower for the age-mismatched vs age-matched cohort before (74.4% vs 82.8%; *P* < .001) and after (72.8% vs 79.9%; *P* = .045) propensity score matching (eFigure 3 in Supplement 1).

Before propensity score matching, among 438 candidates with graft loss within 10 years, 158 (36.1%) received retransplant (94 [6.7%] in the age-matched and 64 [10.5%] in the age-mismatched cohort), including 148 (93.7%) from DBD donors, 6 (3.8%) from living donors, and 4 (2.5%) from DCD donors. After propensity score matching, among 255 recipients with graft loss within 10 years, 79 (31.0%) received retransplant (26 of 526 [4.9%] in the age-matched cohort and 53 of 526 [10.1%] in the age-mismatched cohort).

### Simulation of Broader Sharing of Adolescent Liver Grafts

**Figure 3.**
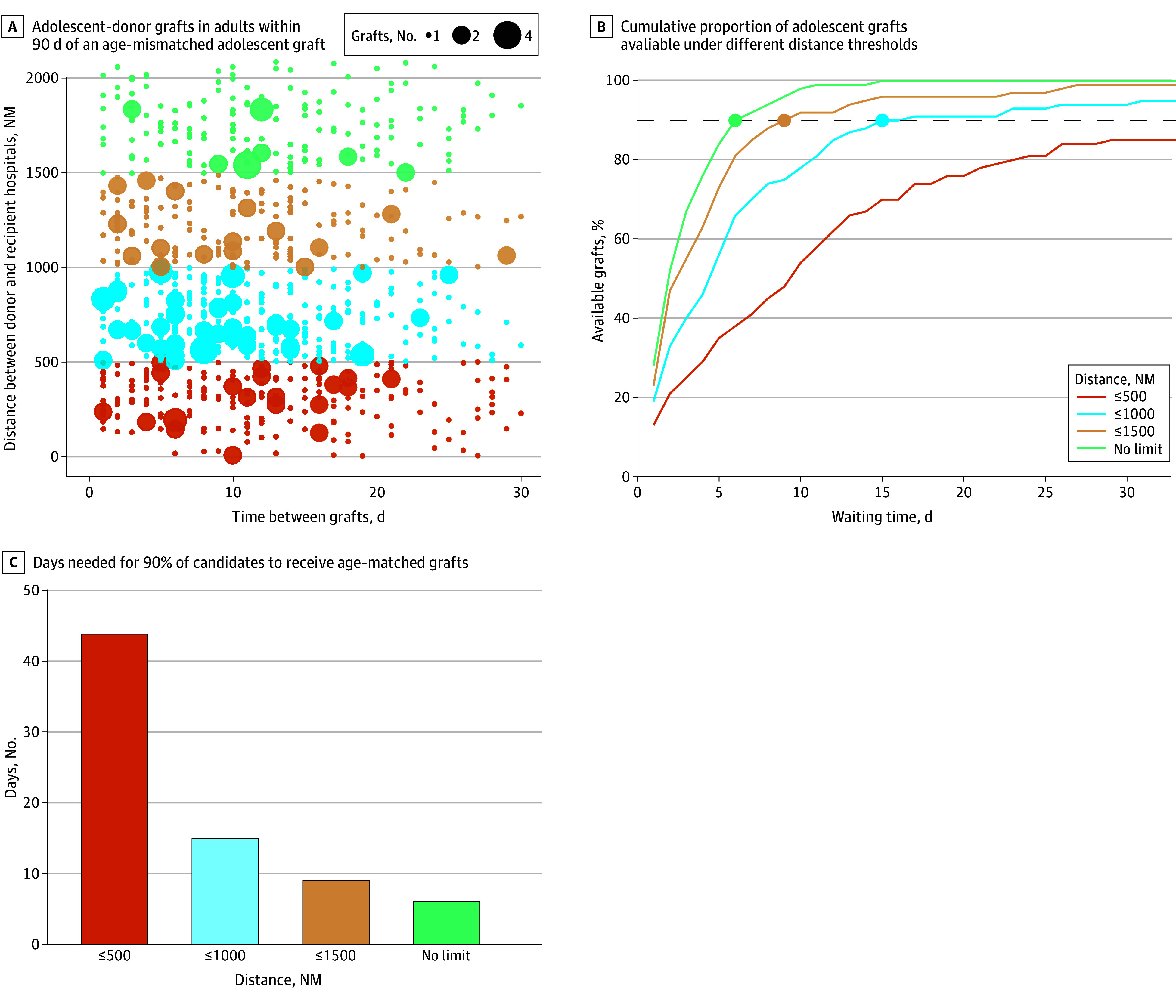
Association of Simulated Broader Sharing of Adolescent Donor Grafts With Waiting Time A, Each dot represents an adolescent donor graft used in an adult recipient within 90 days of an adolescent candidate’s accepting an age-mismatched graft; marker size reflects overlapping grafts. B, Dashed horizontal line represents the day by which 90% of individuals would receive an adolescent graft offer. NM indicates nautical miles.

For each age-mismatched LT after February 4, 2020 (n = 100), the number of days until subsequent LTs using adolescent grafts for adult recipients was calculated. For the first 10 adolescent DBD grafts per candidate, the association between (1) the time interval between the index transplant and each potential adolescent graft and (2) the distance between the index recipient’s hospital and the donor hospital is shown in Figure 3A. Many grafts were transplanted within the first 10 days, but the grafts available within 500 NM were limited. Next, we simulated the cumulative distribution of adolescent graft arrivals to age-mismatched recipients under the 4 distance limits (Figure 3B). We estimated that expanding the allocation radius would shorten the wait for an age-matched liver in adolescents: 90% of patients would receive a matched graft in 15 days at 1000 NM, 9 days at 1500 NM, and 6 days with no distance limit. However, 44 days were needed at the current 500 NM limit (Figure 3C). The 10-year graft survival (72.3%) of 70 grafts that traveled 1000 NM or more was similar to shorter-traveling grafts (70.1% in grafts traveling <500 NM and 74.1% in those traveling 500-999 NM; *P* = .74) (eFigure 5 in Supplement 1).

### Supplementary Analysis

During the study period, 7598 LTs from 6982 adolescent donors were performed: 5401 (71.1%) to adults, 792 (10.4%) to adolescents, and 1405 (18.5%) to children younger than 12 years. We compared the adult recipients with the age-matched adolescent cohort (eTable in Supplement 1). Adults had a median age of 54.0 years (IQR, 46.0-61.0 years) and MELD score of 20.0 (IQR, 13.0-28.0). Donor and recipient BSA were higher in adult transplants, but the donor-recipient BSA difference was small (eFigure 4 in Supplement 1).

## Discussion

This national registry case-control study assessed the association of donor-recipient age discrepancy with posttransplant outcomes of adolescent LTs. Although advanced donor age, such as 70 years or older, is a well-known factor associated with reduced graft survival after LT,^3^ it remains unclear whether grafts from donors aged 30 to 40 years have a similar impact on adolescent recipients. Furthermore, due to a scarce adolescent donor pool, waiting for age-matched donors is not always possible, yet no study to our knowledge has been conducted to estimate the additional wait required for age-matched donors. Our analysis found that the use of age-mismatched donors was more common in sicker candidates and decreased in healthier candidates after 2020. Second, donor-recipient age mismatch was a significant factor associated with graft survival, even 5 years after LT, regardless of pre-LT illness severity. Third, our findings suggest that expanding the allocation radius for adolescent liver grafts may shorten wait times for age-matched offers and reduce reliance on older donors, which are linked to poorer long-term outcomes. To our knowledge, this is the first study analyzing the association of age mismatch with pediatric LT outcomes. Our results indicate a further need to alleviate the use of age-mismatched donors.

Among our study cohort, grafts from age-mismatched donors (median age, 36 years) were used in 30.3% of LTs and were more likely to be used for high-acuity candidates, suggesting persistent shortage of adolescent donor livers for adolescent recipients. LDLT is 1 strategy to address this shortage, with favorable waiting list outcomes reported for pediatric candidates listed at centers with higher LDLT volume.^22^ However, this is not a fundamental solution, nor do we propose increasing pediatric deceased donation. As shown in Figure 1B, more than half of adolescent donor livers are allocated to adults. While this proportion has declined following the 2020 policy change with improved access for home-listed pediatric candidates and lower adolescent waiting list mortality, age-mismatched graft use remains higher than 30% among hospitalized candidates (Figure 1C).^8,9,10^ Although perspectives may vary on how much pediatric prioritization is optimal, it is important to consider the long-term outcomes for adolescent recipients.

While the use of age-mismatched donors might be acceptable if outcomes were comparable, age mismatch was associated with shorter graft survival, regardless of the recipient’s illness severity at transplant. It is important to note that the median donor age for the age-mismatched or older-donor cohort in our analysis was just 36 years, much younger than the threshold for older donors in studies of adult LT, which is mostly between ages 50 and 70 years.^23,24,25,26,27,28^ The reasons for this could be multifactorial. Studies suggest that aging in both mice and humans leads to hepatocyte hypertrophy and a critical breakdown of hepatic zonation homeostasis, thereby increasing the liver’s vulnerability to stress.^29^ When hepatocytes from aged rats were transplanted into a young rat liver enriched with proliferative stimuli, the proliferative capacity was reduced by 50% compared with transplants from young donors.^30^ Moreover, in studies assessing telomere length in biopsies from long-surviving pediatric LT recipients, older donor age was associated with shorter telomere length,^31,32^ and telomere length was shorter than expected for the graft age.^32,33^ Age-mismatched grafts might be insufficient for adolescent candidates with a high metabolic demand and long life expectancy after transplant.^34^ While it might be necessary to use age-mismatched donors in high-acuity cases, there is great variability among transplant centers in the use for nonhospitalized patients (Figure 1D). We believe the use of older grafts for stable adolescent candidates should be minimized.

However, it is difficult to decline an older liver graft in high-acuity cases, because the wait time for an age-matched offer is unpredictable, and we should never compromise short-term waiting list survival by focusing on the long-term graft survival. Efforts to increase donation from pediatric donors and the use of split, living-donor, or DCD grafts might help overcome the donor shortages. However, 52.4% of adolescent livers were allocated to adults in 2024. Among adults, especially those older than 60 years, the benefit of receiving a younger donor liver is limited.^5,6^ Therefore, we believe that further allocation priority through expanding the distance limits for adolescent grafts is an option to reduce the need for age-mismatched transplants. We believe this should be implemented only for hospitalized candidates, as they are the subgroup still requiring age-mismatched grafts. Our analysis estimated that expanding the allocation radius for adolescent grafts to 1000 NM would allow 90% of candidates to receive an age-matched graft with only a 15-day additional wait, compared with 44 days under the current 500-NM policy. Though the longer distance poses concern for longer cold ischemia time, the 10-year graft survival of 70 grafts that traveled 1000 NM or more in our study was similar to shorter-traveling grafts.

### Limitations

This study has several limitations. First, its retrospective registry design cannot fully exclude residual confounding not assessed by the registry, such as granular clinical context in offer acceptance and the urgency not measured by MELD score or ICU admission that relates to the use of age-mismatched donors. Second, some may argue broader allocation is unnecessary given recent declines in adolescent waiting list mortality or the unpredictability of waiting time. However, broader sharing of adolescent grafts would reduce the need for adolescents to accept adult grafts, as the Acuity Circles policy reduced the same need for adolescents who were not hospitalized (Figure 1C). Considering only 2020 adolescent transplants over 23 years, little effect on adult waiting list outcomes is expected. Because long-term LT outcomes have stagnated, prioritizing durable grafts for pediatric candidates may be an essential step toward improving overall long-term results.^35,36^ Third, the registry lacks granular data on pediatric-specific long-term challenges—such as malnutrition, medication adherence, and the transition of care into adulthood—which may also influence outcomes and warrant further investigation.^37,38^

## Conclusions

In this case-control study of a US national cohort of adolescent LT recipients, the use of age-mismatched grafts was associated with inferior graft survival. Although the need for such grafts has decreased for nonhospitalized candidates following recent policy changes, additional efforts, both at the physician level and through allocation policy, are needed to further minimize the use of age-mismatched grafts and improve long-term outcomes for adolescent recipients.

## Notes

Supplement 1. **eTable.** Baseline Donor and Recipient Characteristics Between Adult Recipients of Adolescent Donor Livers and Adolescent Recipients of Age-Matched Livers **eFigure 1.** Kaplan-Meier Survival Curves in Severity Subgroups **eFigure 2.** Kaplan-Meier Survival Curves in Subgroups Stratified by Transplant Year **eFigure 3.** Kaplan-Meier Survival Curves for Overall Survival **eFigure 4.** Distribution of Body Surface Area Between Adult Recipients of Adolescent Donor Livers and Adolescent Recipients of Age-Matched Livers **eFigure 5.** Kaplan-Meier Survival Curves in Subgroups Stratified by Distance Between Donor Hospital and Recipient Hospital

Supplement 2.
Data Sharing Statement
