# Supplementary material for: Donor-Recipient Age Mismatch and Long-Term Graft Outcomes After Adolescent Liver Transplant
Source: JAMA Netw Open. 2026 Jan 7;9(1):e2552779. doi: 10.1001/jamanetworkopen.2025.52779 (PMC12780930; doi:10.1001/jamanetworkopen.2025.52779)
Supplement: Supplement 1. — eTable. Baseline Donor and Recipient Characteristics Between Adult Recipients of Adolescent Donor Livers and Adolescent Recipients of Age-Matched Livers eFigure 1. Kaplan-Meier Survival Curves in Severity Subgroups eFigure 2. Kaplan-Meier Survival Curves in Subgroups Stratified by Transplant Year eFigure 3. Kaplan-Meier Survival Curves for Overall Survival eFigure 4. Distribution of Body Surface Area Between Adult Recipients of Adolescent Donor Livers and Adolescent Recipients of Age-Matched Livers eFigure 5. Kaplan-Meier Survival Curves in Subgroups Stratified by Distance Between Donor Hospital and Recipient Hospital [file jamanetwopen-e2552779-s001.pdf]

Supplementary Online Content

Nakayama T, Jensen AR, Attia A, et al. Donor-recipient age mismatch and long-term graft outcomes after adolescent liver transplant. *JAMA Netw Open*. 2026;9(1):e2552779. doi:10.1001/jamanetworkopen.2025.52779

eTable. Baseline Donor and Recipient Characteristics Between Adult Recipients of Adolescent Donor Livers and Adolescent Recipients of Age-Matched Livers
eFigure 1. Kaplan-Meier Survival Curves in Severity Subgroups
eFigure 2. Kaplan-Meier Survival Curves in Subgroups Stratified by Transplant Year
eFigure 3. Kaplan-Meier Survival Curves for Overall Survival
eFigure 4. Distribution of Body Surface Area Between Adult Recipients of Adolescent Donor Livers and Adolescent Recipients of Age-Matched Livers
eFigure 5. Kaplan-Meier Survival Curves in Subgroups Stratified by Distance Between Donor Hospital and Recipient Hospital

This supplementary material has been provided by the authors to give readers additional information about their work.

eTable. Baseline donor and recipient characteristics between adult recipients of adolescent donor livers and adolescent recipients of age-matched livers.

	Adolescent recipients	Adult recipients	P value
N	1408	5401	
Graft type and size mismatch			<0.001
Reduced/split graft	95 (6.7)	577 (10.7)	
Whole liver, small size	143 (10.2)	640 (11.8)	
Whole liver, normal size	965 (68.5)	4009 (74.2)	
Whole liver, large size	205 (14.6)	175 (3.2)	
Donor age	16.0 [13.0, 17.0]	16.0 [14.0, 17.0]	0.54
Donor sex			<0.001
Female	510 (36.2)	1661 (30.8)	
Male	898 (63.8)	3740 (69.2)	
Donor/recipient sex mismatch, yes	680 (48.3)	2344 (43.4)	0.001
Donor height, cm	166.7 [154.9, 175.0]	170.2 [163.0, 178.0]	<0.001
Donor weight, kg	61.2 [49.9, 72.0]	67.1 [57.5, 77.9]	<0.001
Donor BSA, m2	1.7 [1.5, 1.9]	1.8 [1.6, 1.9]	<0.001
Cold ischemia time	6.8 [5.2, 8.6]	6.4 [5.0, 8.3]	<0.001
Recipient age	15.0 [13.0, 16.0]	54.0 [46.0, 61.0]	<0.001
Recipient sex			<0.001
Female	754 (53.6)	2377 (44.0)	
Male	654 (46.4)	3024 (56.0)	
Recipient height, cm	160.0 [152.4, 168.0]	170.2 [162.6, 177.8]	<0.001
Recipient weight, kg	55.3 [45.0, 66.2]	78.0 [65.3, 91.6]	<0.001
Recipient BSA, m2	1.6 [1.4, 1.7]	1.9 [1.7, 2.1]	<0.001
Diagnosis, pediatric			
BA	134 (9.5)		
Acute liver failure	180 (12.8)		
Metabolic	168 (11.9)		
Other cholestatic	253 (18.0)		
Others	673 (47.8)		
Diagnosis, adult			
ALD		1099 (20.3)	

© 2026 Nakayama T et al. *JAMA Network Open*.

Autoimmune		713 (13.2)	
HCC		474 (8.8)	
HCV		1076 (19.9)	
liver failure, acute		356 (6.6)	
MASLD		450 (8.3)	
others		1233 (22.8)	
MELD	16.0 [11.0, 22.0]	20.0 [13.0, 28.0]	<0.001
Status 1	320 (22.7)	451 (8.4)	<0.001
Pretransplant condition			0.002
ICU	250 (17.8)	838 (15.5)	
Hospital	187 (13.3)	912 (16.9)	
Home	971 (69.0)	3651 (67.6)	
History of previous transplant, yes	149 (10.6)	440 (8.1)	0.004
Waiting days	68.0 [14.0, 221.2]	71.0 [11.0, 258.0]	0.83

Note: continuous variables: median [IQR]; categorical variable: number (%).

Abbreviations: BSA, body surface area; HCC, hepatocellular carcinoma; HCV, hepatitis C virus; ICU, intensive care unit; MASLD, metabolic dysfunction-associated steatotic liver disease; liver MELD, Model for End-Stage Liver Disease.

© 2026 Nakayama T et al. *JAMA Network Open*.

eFigure 1. Kaplan-Meier survival curves in severity subgroups.

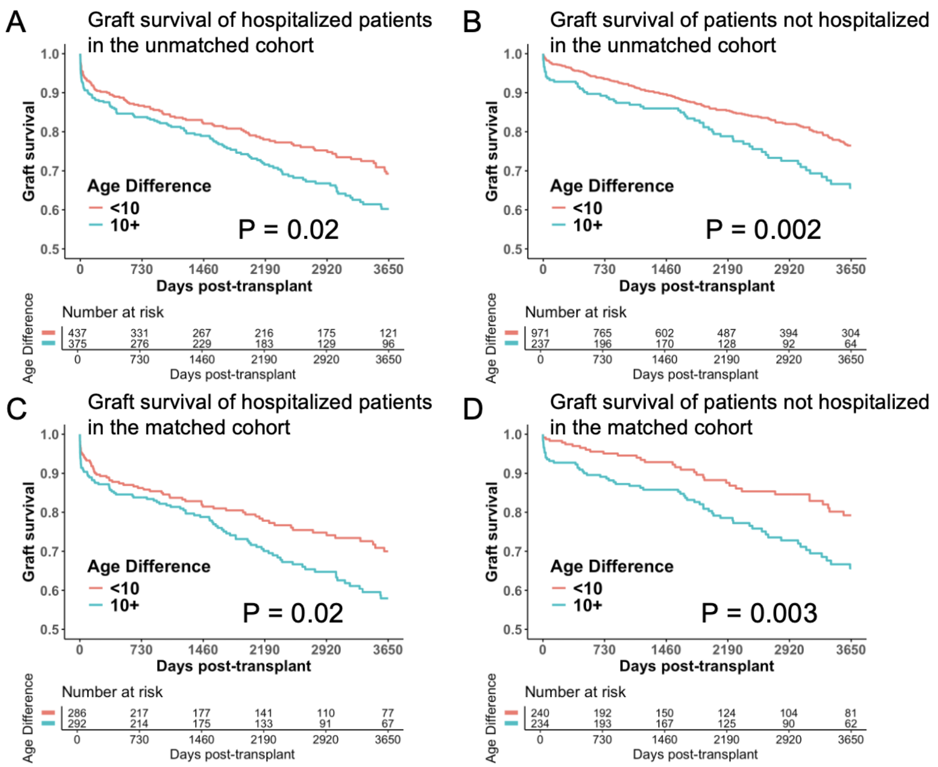

(A) Kaplan-Meier survival curve in candidates hospitalized before LT in the unmatched cohort. (B) Kaplan-Meier survival curve in candidates not hospitalized before LT in the unmatched cohort. (C) Kaplan-Meier survival curve in candidates hospitalized before LT in the matched cohort. (D) Kaplan-Meier survival curve in candidates not hospitalized before LT in the matched cohort.

Abbreviations: LT, liver transplantation.

© 2026 Nakayama T et al. *JAMA Network Open*.

eFigure 2. Kaplan-Meier survival curves in subgroups stratified by transplant year

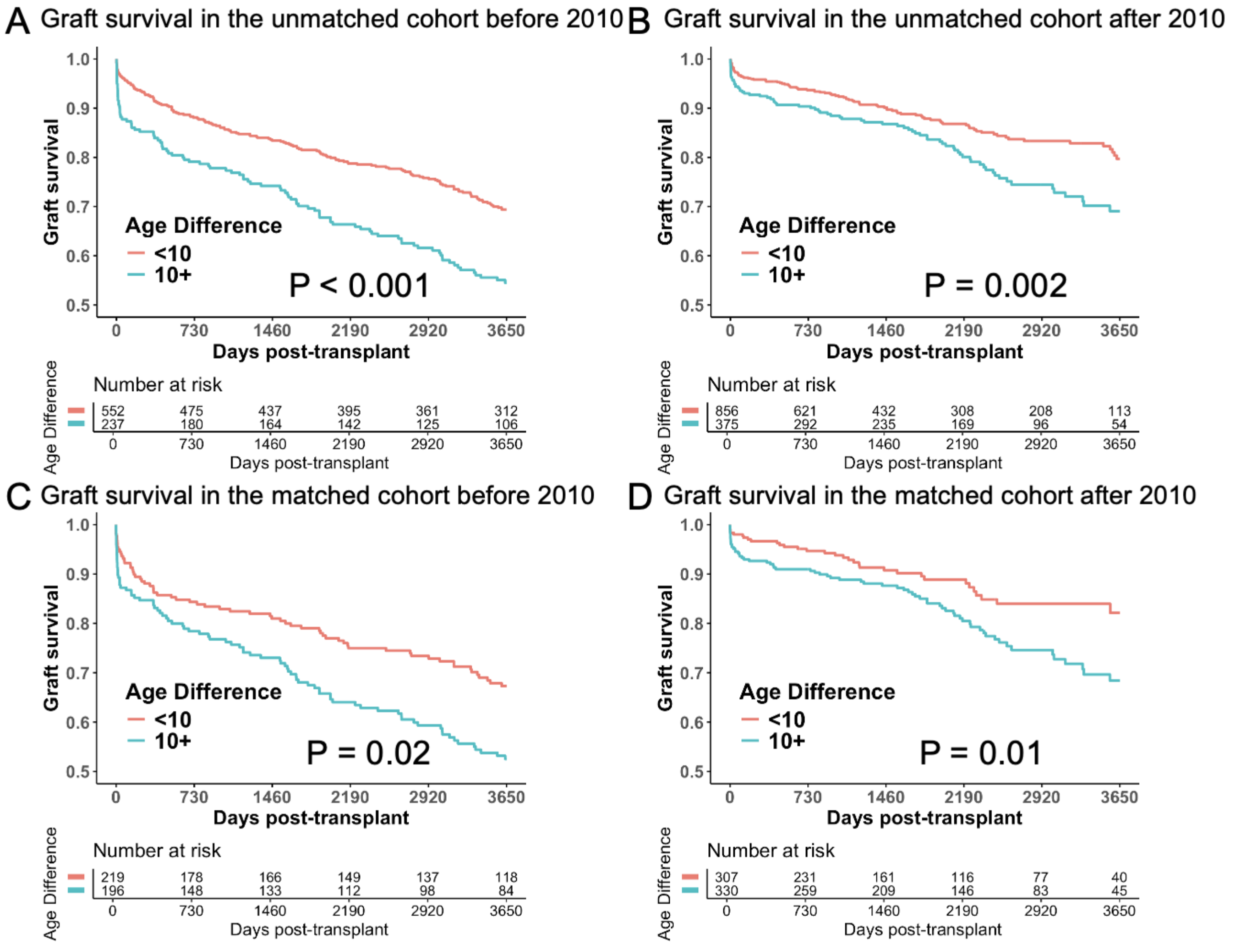

(A) Kaplan-Meier survival curve in the unmatched cohort for LT performed between 2002 and 2010. (B) Kaplan-Meier survival curve in the unmatched cohort for LT performed between 2011 and 2024. (C) Kaplan-Meier survival curve in the unmatched cohort for LT performed between 2002 and 2010. (D) Kaplan-Meier survival curve in the matched cohort for LT performed between 2011 and 2024.

eFigure 3. Kaplan-Meier survival curves for overall survival.

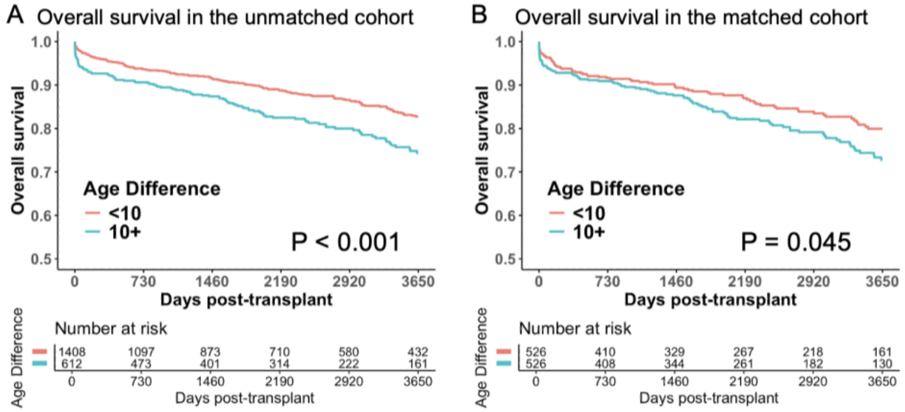

(A) Overall survival in the unmatched cohort.

(B) Overall survival in the matched cohort.

© 2026 Nakayama T et al. *JAMA Network Open*.

eFigure 4. Distribution of body surface area between adult recipients of adolescent donor livers and adolescent recipients of age-matched livers.

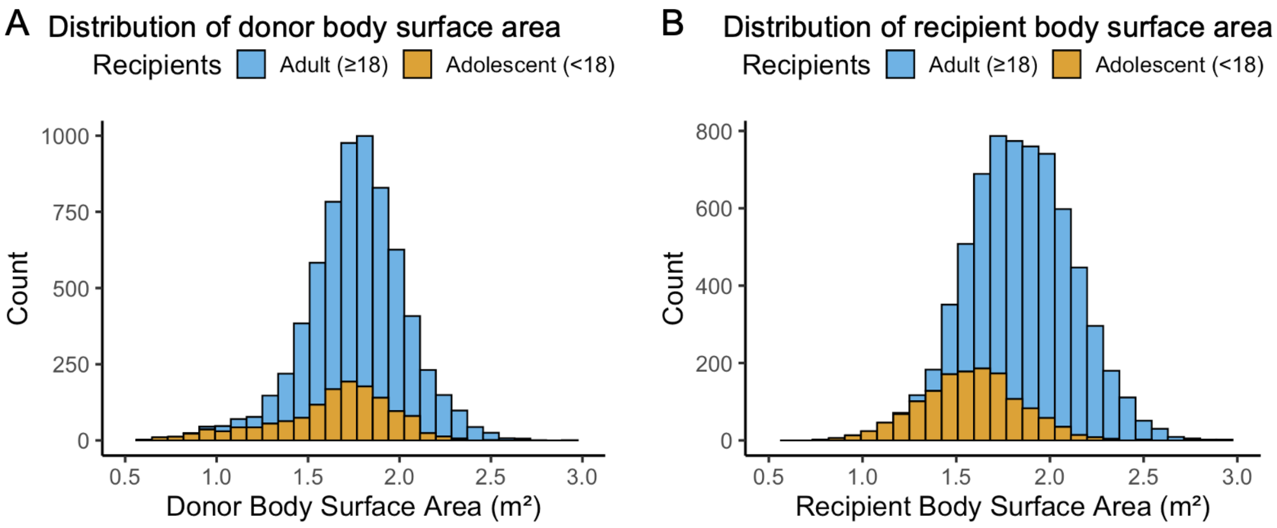

(A) Distribution of donor body surface area.

(B) Distribution of recipient body surface area.

© 2026 Nakayama T et al. *JAMA Network Open*.

eFigure 5. Kaplan-Meier survival curves in subgroups stratified by distance between donor hospital and recipient hospital.

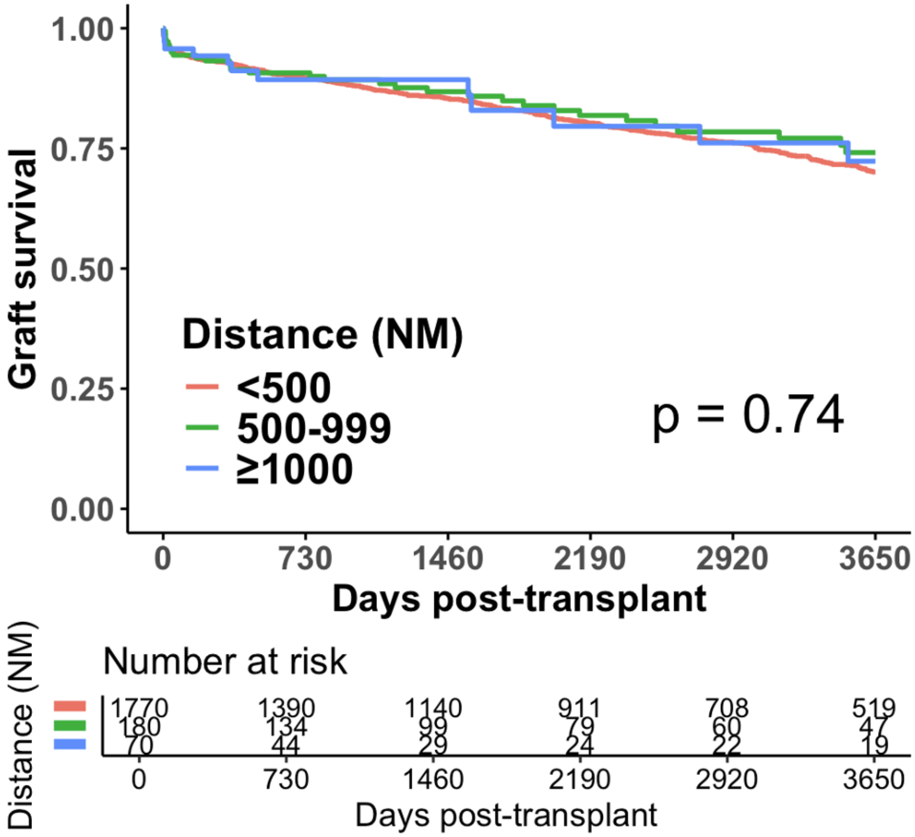

© 2026 Nakayama T et al. *JAMA Network Open*.
