# Supplementary material for: Donor-Recipient Age Mismatch and Long-Term Graft Outcomes After Adolescent Liver Transplant
Source: JAMA Netw Open. 2026 Jan 7;9(1):e2552779. doi: 10.1001/jamanetworkopen.2025.52779 (PMC12780930; doi:10.1001/jamanetworkopen.2025.52779)
Supplement: Supplement 2. — Data Sharing Statement [file jamanetwopen-e2552779-s002.pdf]

Data Sharing Statement

Nakayama. Donor-Recipient Age Mismatch and Long-Term Graft Outcomes After Adolescent Liver Transplant. *JAMA Netw Open*. Published January 07, 2026.
doi:10.1001/jamanetworkopen.2025.52779

Data
Data available: Yes
Data types: Deidentified participant data, Data dictionary
How to access data: This study utilized data from the UNOS Standard Transplant Analysis and Research (STAR) file, a publicly available database. Access to the STAR file is granted by UNOS upon reasonable request.
When available: With publication

Supporting Documents
Document types: None

Additional Information
Who can access the data: Data requests can be made directly to the United Network for Organ Sharing by any researchers.
Types of analyses: for any purpose
Mechanisms of data availability: After approval by the United Network for Organ Sharing with a signed data access agreement
Any additional restrictions: None
